# Progress in research on gut microbiota in ethnic minorities in China and consideration of intervention strategies based on ethnic medicine: A review

**DOI:** 10.3389/fcimb.2022.1027541

**Published:** 2022-10-18

**Authors:** Rong Chen, Zhong-Yu Duan, Xiao-Hua Duan, Qing-Hua Chen, Jin Zheng

**Affiliations:** ^1^ School of Ethnic Medicine, Yunnan University of Chinese Medicine, Kunming, China; ^2^ Yunnan Key Laboratory of Dai and Yi Medicines, Yunnan University of Chinese Medicine, Kunming, China

**Keywords:** gut microbiota, ethnicity, ethnic medicine, China, review

## Abstract

One of the variables affecting gut microbiota is ethnicity. There are 56 ethnic subgroups in China, and their intestinal flora differs. A wealth of medical resources has also been produced by the presence of numerous ethnic minorities. In this study, we reviewed the pertinent literature on the intestinal flora of ethnic minorities in China and abroad using the CiteSpace visualization software, and we used bibliometric techniques to find the most widely prescribed medications for preventing and treating endemic diseases in ethnic minorities. Based on the gut microbiology of minority populations, we suggest that by comprehensive development involving literature, experimental, and clinical research, the pharmacological action mechanisms for interventions in endemic diseases can be drawn from ethnic medicine. This point of view has not been discussed before and will offer a fresh perspective on the creation and application of ethnic medications as well as a fresh method for the management of prevalent diseases in ethnic communities.

## Introduction

The gut microbial community is the largest and most diverse in terms of species in the complex ecosystem that makes up the human microbiome (Tan et al., 2020). More than 5 million non-redundant microbial genes encoding up to 20,000 biological activities associated with life in the gut environment have been found through comprehensive metagenomic research. Designing interventions to enhance symbiosis and fight disease will be made easier with a greater understanding of the contributions that microbial symbionts make to host health (Robles-Alonso and Guarner, 2013). Approximately 90% of all illnesses that affect people have some connection to the bowel, and intestinal control is now understood to be the foundation and entry point for many illnesses (Che et al., 2022). The underlying causes of numerous illnesses have been connected thanks to the ongoing advancements in gut microbiota research. The restoration of the gut’s unbalanced microbiota is also emerging as a potential alternate course of action (Li et al., 2022). The bacteria that live in the human intestine are collectively referred to as the gut microbiota. These microorganisms actively participate in the anabolism and catabolism of materials, contributing to the maintenance of a favorable environment in the colon through their physiological activity (Che et al., 2022). Many investigations into the gut microbiota composition demonstrated that a variety of factors significantly influence individual differences in terms regarding the gut microbiota’s composition and diversity (Turnbaugh et al., 2009; Rothschild et al., 2018). These variables include the host genome composition, the geography, the diet, and the way of life (Turnbaugh et al., 2009; Yatsunenko et al., 2012). Studies have revealed that a number of microbial taxa, gene families, and metabolic pathways were strongly related with ethnic background. The term “ethnicity” probably refers to a wide range of intricate interactions between internal and external factors that have an impact on the composition of the gut microbiota. A growing body of research pointing to microbial variations by ethnicity supports this (Deschasaux et al., 2018; Yang et al., 2019). However, this can be attributed to differing nutritional intake and other behaviors shared by those subpopulations of people from different geographical areas (De Filippo et al., 2010; Yatsunenko et al., 2012).

The use of stool therapy dates back to over 1700 years ago in traditional Chinese medicine (Zhang et al., 2018). FMT was first described as an adjuvant treatment for patients with antibiotic-associated diarrhea by (Eiseman et al., 1958), ushering in the contemporary age (Li et al., 2022). The makeup of gut bacteria and the transformation of active metabolites are significant aspects of traditional Chinese medicine (TCM) (Li et al., 2022). From the standpoint of TCM, According to Chinese medicine, the human body, with its tissues and organs, as well as the surroundings, nature, and way of life, are one cohesive whole. People’s many organs are interrelated and affect the whole-body physiology in a way that prevents them from functioning independently. The relationship between TCMs and intestinal flora is reciprocal: on the one hand, TCMs have the ability to control the composition and metabolic activity of the flora by selectively blocking or supporting the growth of various intestinal microbes, thereby improving human health. On the other hand, TCMs will be metabolized by the gut flora, which could result in harmful metabolites being produced or their efficacy being increased (Che et al., 2022).

Despite being regarded as a subset of TCM, ethnomedicine has been accorded a relatively independent standing under the People’s Republic of China’s Law on Traditional Chinese Medicine since 2017 (Li et al., 2020). China is a diverse nation with 56 different nationalities, 55 of which are recognized by the government as ethnic minorities in 18 different provinces. Each ethnic minority has its own traditional medicine, which varies slightly in theory and application from TCM. Examples include the Tibetans, Mongols, Uygurs, Dai, Yi, and Miao. Thus, ethnomedicine is the practice of traditional medicine that is informed by each ethnic minority’s medical philosophy and real-world experience (Zhu et al., 2016). Many ethnic minorities in China remain adhere to the medical traditions established in their communities, despite the widespread use of modern medicine in the country. Numerous well-known medications, like artemisinin (Ma et al., 2020) and Yunnan Baiyao (Yao et al., 2021) were discovered by ethnic minorities through such medical practices. Ethnic medicine places a strong emphasis on how geography, environment, lifestyle, and ethnicity all affect health and disease, which is in line with gut microbiota. Finding new medications from ethnomedicine to treat diseases based on gut bacteria is thus a novel approach.

**Figure 1:**
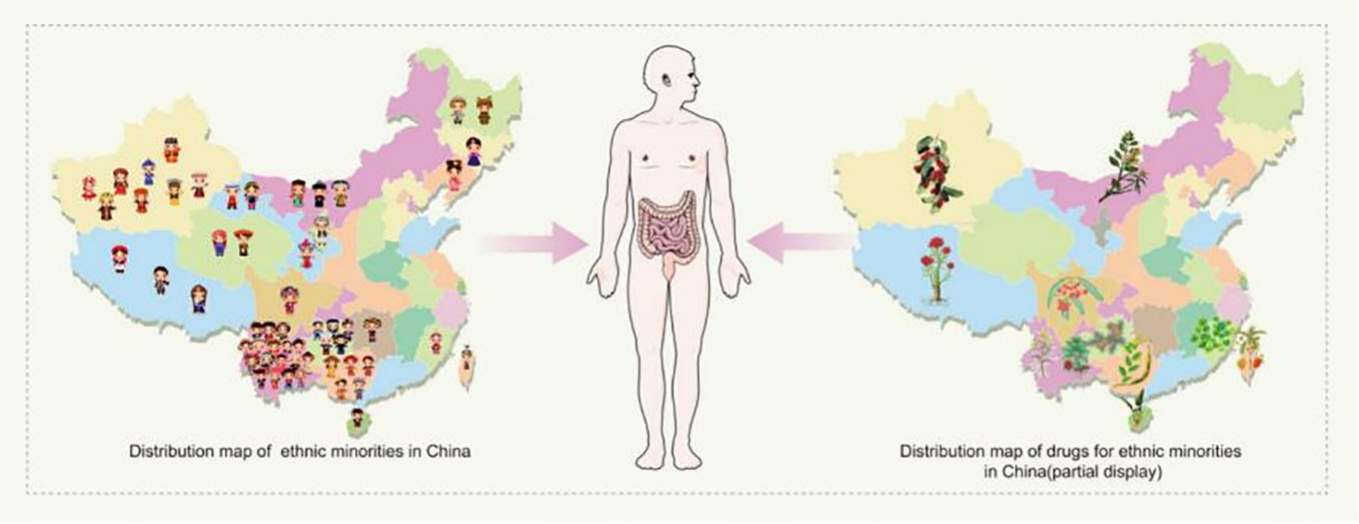
China’s Ethnic Minorities and Ethnic Minority Drug Distribution Map (partial display).Intervention of intestinal microorganisms of populations in ethnic areas through ethnomedicine for the treatment of endemic diseases in ethnic populated areas.

This paper summarizes the role of gut microbes in ethnic health and disease disparities, along with tactics used in ethnic medicine to treat prevalent diseases affecting minorities, using China as an example (**Figure 1**).

## Global research on the gut microbiota of ethnic minorities

The human gut microbiota has regularly been shown to be strongly influenced by ethnicity (Dwiyanto et al., 2021). Numerous microbial species make up the human gut microbiota, which might change depending on the chronic diseases that cause health inequalities and disproportionately affect ethnic minorities. However, studies on this subject have not produced generalizations that can be repeated, and the impact of ethnicity on gut microbiota is still largely unknown (Brooks et al., 2018).

**Figure 2:**
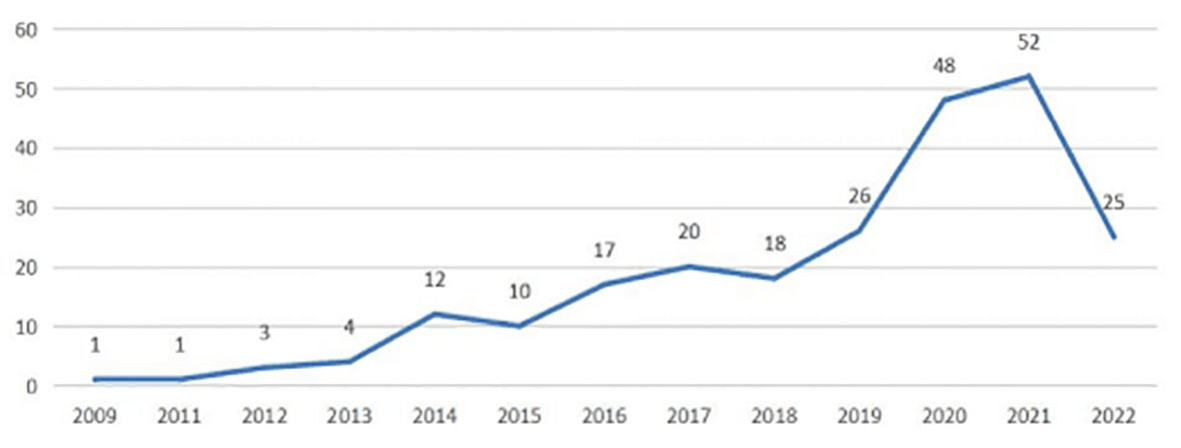
Annual statistics of published papers. The abscissa represents time, and the ordinate represents the number of papers.

**Figure 3:**
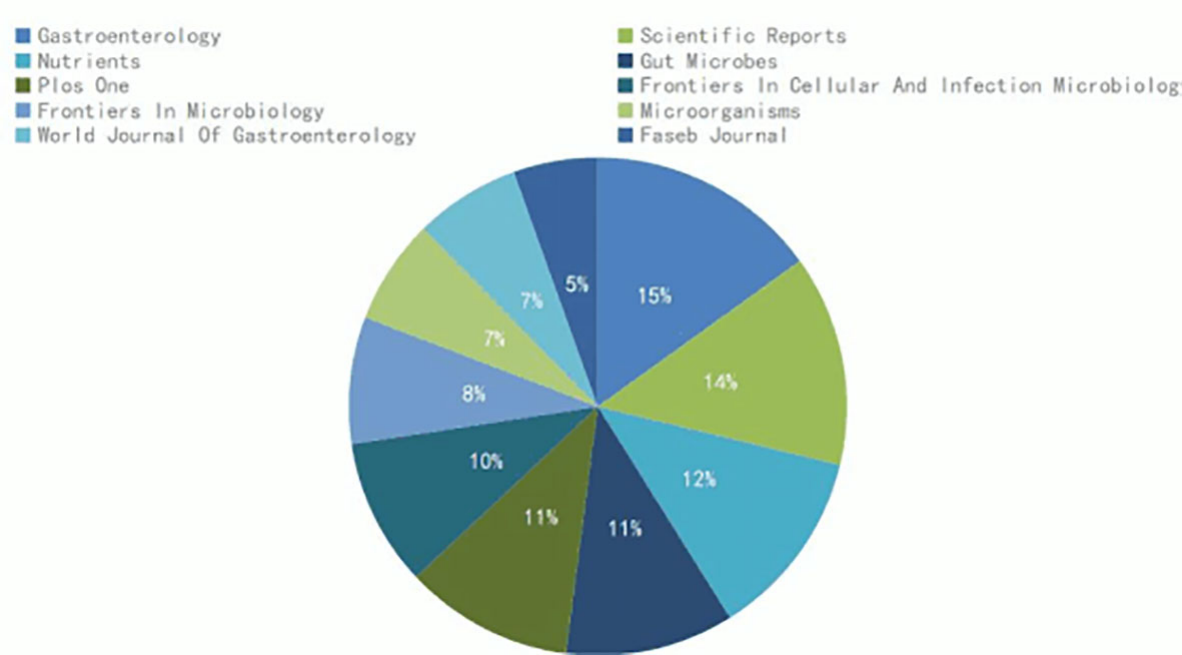
Proportions of papers published in specific journals. The larger the area, the more articles be posted.

**Figure 4:**
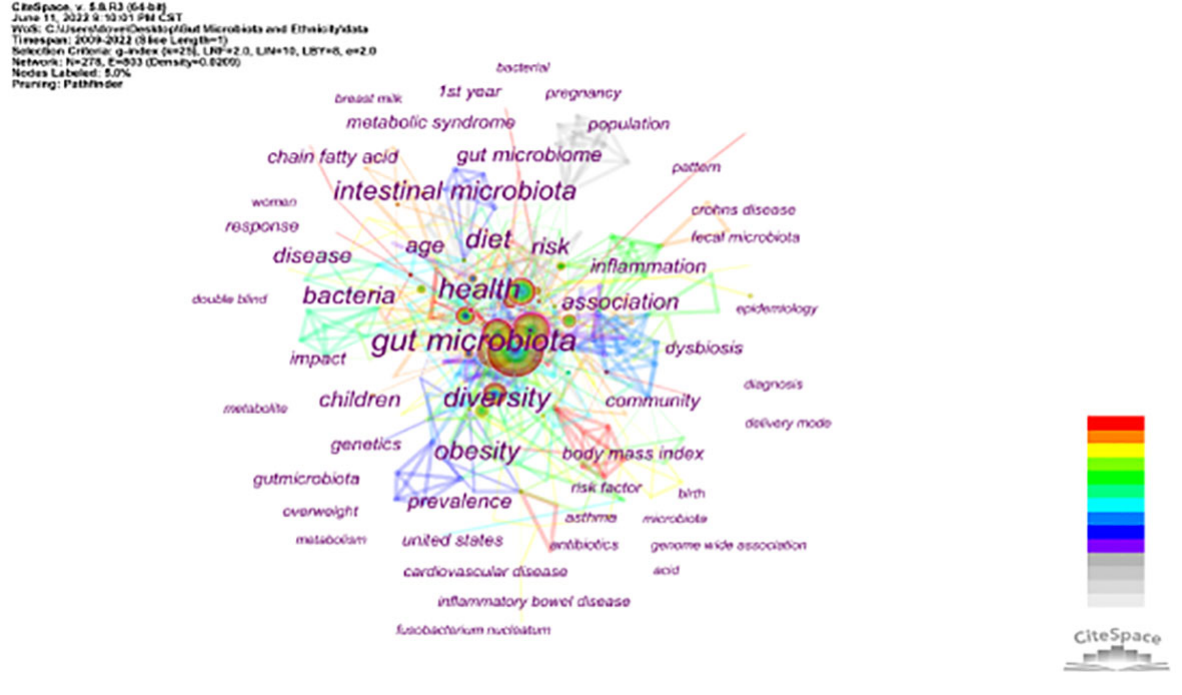
Keyword map for research on “Gut Microbiota” and “Ethnicity” conducted from 1984 to 2022. The larger the keyword node, the stronger its neutrality. The line of nodes represents the co-occurrence relationship, the thickness of the line.indicates the intensity of co-occurrence.The color from gray to red indicates the time from early to recent.

**Figure 5:**
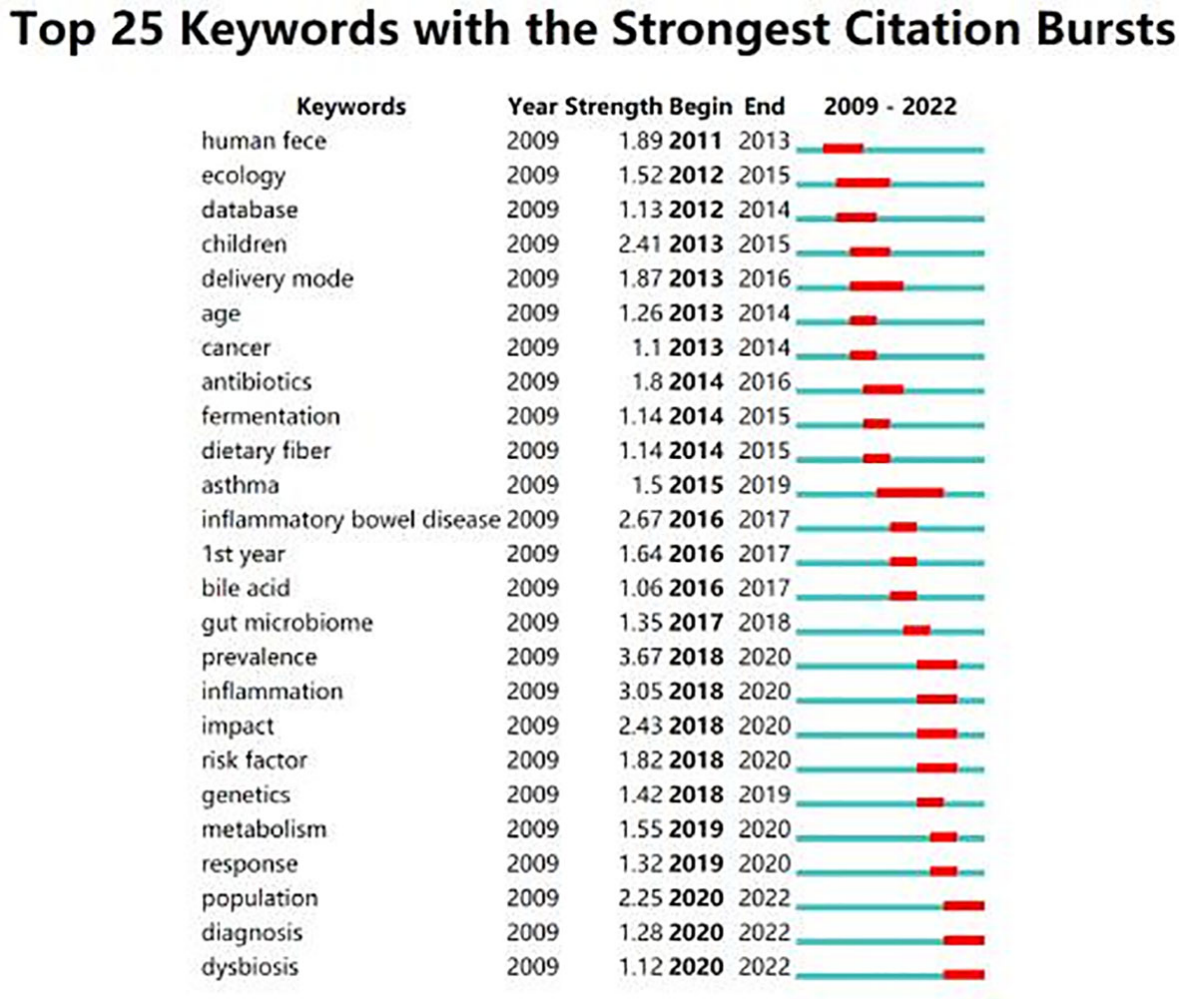
Keywords with the strongest citation bursts. The keywords with high frequency change rate and fast growth rate that can reflect the frontiers of research based on minority gut microbes between 2009-2022.

**Figure 6:**
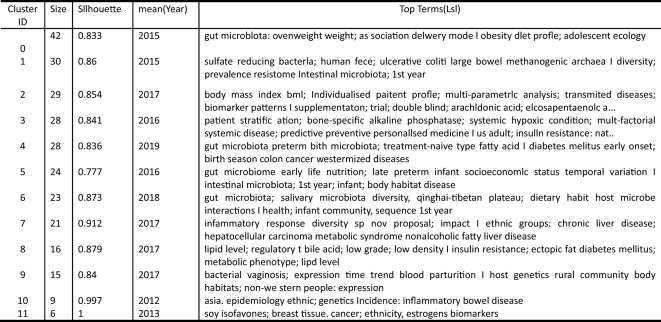
Keyword clustering. The smaller the number, the more keywords are included in the clusters, and each cluster is composed of multiple closely related words.

**Figure 7:**
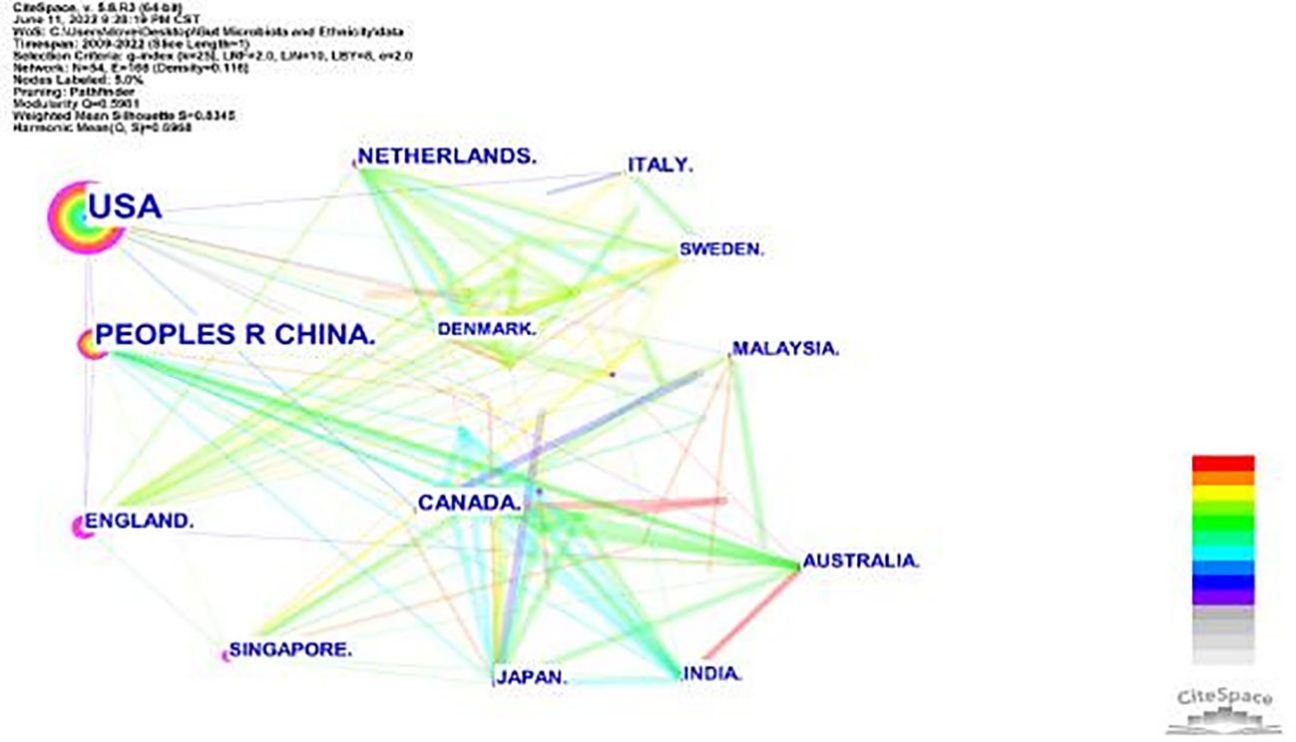
National co-occurrence network diagram. The larger the keyword node, the stronger its neutrality. The line of nodes represents the co-occurrence relationship, the thickness of the line indicates the intensity of co-occurrence. The color from gray to red indicates the time from early to recent. The figure shows the collaborative network between countries for journal articles related to minority gut microbes.

The creation of therapies for focused microbiota modification can be made easier with the help of ethnicity-specific microbial signatures (Dehingia et al., 2019). The core collection database of the Web of Science (WOS) was utilized as the database source, and the world-wide variations in gut microbiota due to ethnicity were examined using CiteSpace 5.8 software. A knowledge map was used to examine the pertinent publications, and the research hotspots in this area were compiled. To represent publications, sources, countries, and keywords, we created a variety of visual maps. Studies indexed from the beginning of the database until August 1, 2022 were searched for in the WOS Core Collection. We searched for studies using the terms “Gut Microbiota” and “Ethnicity,” and the searches turned up a total of 237 papers on the topic. Since the initial article, “Are Urinary Metabolic Differences Related to Host Microbial Interactions Identical in Different Ethnic Groups with Crohn’s Disease?,” (Walker, David G et al., 2009) was published, we have discovered that the number of papers in the last three years has drastically increased in the journal Gastroenterology (impact factor [IF] = 33.383 in 2009), showing that the connections between race and gut microbiota have gained research attention and are currently a research hotspot (**Figure 2**). The sources for these publications are shown in the proportional journal source chart in **Figure 3**. Knowledge has a bigger impact when the impact factor is higher. Gut microbiota and ethnicity-related articles occasionally appear in publications with high impact factors (*Nature, Cell*), but more frequently in journals with medium impact values (*Journal of Microbiological Methods*). These studies, which highlight the scientific potential of this area of inquiry, are also published in specialized journals (*Frontiers in Cellular and Infection Microbiology; Frontiers in Microbiology*) (**Figure 3**). The article’s main summary is its list of keywords. According to a keyword analysis, the keywords “health,” “diet,” “fat,” “risk,” and “disease” were frequently used. Following the literature, we discovered that the debate of ethnic gut microbiota is centered on health and disease. Due to varying gut microbiota, different ethnic groups suffer from various ailments. In **Figure 4** Burst keywords are those that have been used a lot recently. Burst keywords—indicators of frontier themes across time—were recorded using CiteSpace (Huang et al., 2020). “inflammation”, “metabolomics”, and “genetics “were discovered to be powerful keywords through the analysis of keyword emergence, showing the addition of more disciplines to the study of gut microbiota and the enlargement of its research content. The mechanism of pharmacological intervention in diverse gut microorganisms for illness treatment is typically the focus of research (**Figure 5**). The term cluster may also represent areas of active research. “Qinghai “and “Tibetan” merit our attention in this study (**Figure 6**). According to the examination of national co-occurrence, “Singapore”, the “United States”, “China”, the “United Kingdom”, “India” have all conducted more research on this subject and have developed collaborative partnerships with one another (**Figure 7**).

From 1984 to 2022, research interest in “gut microbiota” and “ethnicity” increased, according to an analysis of the publications, sources, keywords, and countries linked to these topics. Every year since 2009, there have been more studies published, and over the last three years, the growth rate has been tremendous. Although several journals with high impact factors have also published pertinent articles, the impact factors of the periodicals are only moderate (*Nature, Cell*). Additionally, China, the United States, and other multi-ethnic nations are the primary locations for study on “gut microbiota and ethnicity.” From the viewpoint of keyword analysis, a growing amount of attention is being paid to mechanism analyses in various ethnic groups, settings, and diseases. Being a multi-ethnic nation, China’s academics have recently started to focus on the connection between endemic diseases affecting ethnic minorities like the Qinghai, Tibetans, and Mongolians, and the gut microbiota. In summary, numerous research have established a strong connection between “ethnicity” and “gut microbiota.” Additionally, this is a subject that has a lot of potential and worth and will benefit numerous patients.

## The state of gut microbiota research in China’s ethnic minorities

As a vast country, China contains nearly 20% of the world population, including the majority Han and 55 other minority ethnic groups living in their local regions, which show endemic characteristics in genetics (Chu et al., 1998), lifestyle, diet, culture given the tight relationship between the gut microbiota and these factors (De Filippo et al., 2010; Yatsunenko et al., 2012; Tyakht et al., 2013), the diverse ethnic origins of Chinese people offer an excellent opportunity to understand the diversity, variabilities, and commonalities of gut microbiota (Zhang et al., 2015). Thirteen articles about “ethnicity” and “gut microbiota” in WOS were gathered in total. We summarized the current status of research on gut microbiota in Chinese ethnic minorities and found that the huge difference in the incidence of Type 2 diabetes between Mongolians and Han. Because *Fastidiosipila and Barnesiella* were most closely related to diabetes, and these colonies are specific gut microorganisms of Mongolians. Tibetan gut microbes(*Candida, Fusarium, Zopfiella)* were associated with a high prevalence of cardiovascular disease (**Table 1**).

**Table 1:** A total of 13 articles about “gut microbiota” “China” “ethnicity” in WOS were collected.

No.	Ethnicity	Research topics/diseases	Gut microbiota	Conclusion
1	Li	Human-milk	Acinetobacter, Cupriavidus, Staphylococcus petrasii	The ethnic origin of individuals may be an important factor to consider in human milk microbiome research and its potential clinical significance during the perinatal period in ethnic-diverse societies, even within a small geographic scale. (Xie et al.,2022)
2	Zang, Naxi, Han, Lisu, Yi, and Bai	HPV infection	Lactobacillus, Gardnerella, Lactobacillus,	Lactobacillus dominated most of the vaginal samples, was decreased in HPV-positive samples, and differed among different ethnic groups (Liu et al., 2022)
3	Zhuang	Septicaemia	Roseburia, Bifidobacterium, Faecalibacterium Ruminococcus, Anaerostipe, Clostridium,	This study demonstrated significant differences in the gut microbiome between Zhuang ethnic patients with sepsis and healthy individuals. In the future, it is necessary to determine whether such alterations are the cause or consequence of sepsis. (Yu et al., 2022)
4	Tibetan; Hui;Miao; Uygur; Naxi; Mongolian; Bai	Geography Ethnicity	Prevotella, Bacteroides,	the gut microbiota of Han Chinese and ethnic minority groups from the same sites was more alike than that of the same ethnic minority groups from different sites. (Lu et al., 2021)
5	Tibetan	Oral microbial structure	Candida,Fusarium, Zopfiella,Streptococcus, Veillonella, Rothia	there are significant differences in oral microbial structure and metabolic characteristics and trophic modes among Tibetan and Han population living at different altitudes. (Dong et al., 2021)
6	Han; Mongolian	Type 2 diabetes	Flavonifractor, Alistipes, Prevotella, Oscillibacter, Clostridium XlVa	The composition of diabetes-related bacteria were significantly different among the different ethnic groups (Li et al., 2021)
7	Tibetan	Tibetan Oral Bacteria and Fungi Microbiota	Candida, Fusarium, Zopfiella, Streptococcus, Veillonella and Rothia	There are significant differences in oral microbial structure and metabolic characteristics and trophic modes among Tibetan and Han population living at different altitudes (Dong et al., 2021)
8	Hui	Oral and gut microbiota	Streptococcus, Moraxella, Porphyromonas	The consumption of seafood, poultry, sweets and vegetables was significantly correlated with multiple oral microbiotas. (Liu et al., 2021b)
9	Mongolian;Tibetan; Uygur	Gut microbiota	Prevotella and Bacteroides	Both geographical location and ethnicity were major factors (Lin et al., 2020)
10	Tibetan	Coronary heart disease	Blautia, Desulfovibrio, and Succinivibrio	Coronary heart disease (CHD) is closely related to gut microbiota, which m ay be significantly affected by ethnicity and the environment. (Liu et al., 2020)
11	Tibetan;Hui	Diet;ethnicity	Proteobacteria	Ethnic Differences Shape the Alpha but Not Beta Diversity of Gut Microbiota from School Children in the Absence of Environmental Differences (Liu et al., 2021a)
12	Tibetan	Altitude and ethnicity on human gut microbiota	Firmicutes, Bacteroidete	Genetic and dietary factors may also explain the different microbiota compositions between Tibetan and Chinese Han. (Li and Zhao, 2015)
13	Bai, Kazakh, Mongol, Tibetan, Uyghur and Zhuang	Ethnicities/geography	Phascolarctobacterium,Klebsiella	A phylogenetically diverse core of gut microbiota at the genus level may be commonly shared by distinctive healthy populations as functionally indispensable ecosystem service providers for the hosts (Zhang et al., 2015)

The following is a summary of the content.

Several research projects on the gut microbiota of Chinese ethnic minorities were among them. More studies on the gut bacteria of Tibetans, Mongolians, Hui, and Zhuang were indicated by the study material, with publications on the Tibetans’ gut microorganisms appearing the most frequently. Some research focused on the variations in the prevalence of the same disease among various ethnic groups, which are brought on by various gut bacteria among ethnic minorities (Liao et al., 2018; Li et al., 2021; Liu et al., 2021a). Other research have demonstrated a strong correlation between Tibetans’ gut bacteria and the high prevalence of endemic diseases in ethnic minority locations, such as coronary heart disease (Ma et al., 2021). Numerous research on the gut microbiota of ethnic minorities also take into account geography and way of life in addition to ethnicity (Liu et al., 2021b).

## Intervention techniques in Chinese minority medicine that target intestinal microorganisms

Traditional Chinese medicines, traditional medicines used by other ethnic groups, and widely utilized folk medicines for disease prevention or treatment are the most common traditional medicines in China. These treatments include traditional medicines utilized by ethnic groups, which are referred to as “ethnomedicines of Chinese ethnic minorities,” meaning that they are administered in accordance with each Chinese ethnic minority’s individual traditional medicine theory or clinical experience (Zhu, et al., 2016). Ethnomedicines have their roots in the traditional treatments used by ethnic minorities and reflect their view of life, health, and disease. Their commitment to living in harmony with nature. Although this knowledge departs greatly from contemporary scientific standards, some of its ideas are reasonable, practicable, and frequently accepted by most users (Huai et al., 2000). According to ethnomedicine, each person’s life and death are directly correlated with their ethnicity, environment, and way of life. Intestinal bacteria determine the diseases to which a person is predisposed, and the remedy for those diseases can be found in the medicines that develop around him. The therapy of sickness is the restoration of harmony between the inside and the outside of the body. Numerous reports have supported this opinion (Zhu et al., 2022).

According to a study of other publications, ethnic minorities in China have a diverse gut flora from the Han population. Endemic diseases in each ethnic group are correlated with their intestinal microbiota, and this difference is the starting point for disease occurrence and therapy. There is a wealth of knowledge about drug usage among racial and ethnic minorities in the history of endemic illness prevention and treatment. How may endemic diseases, ethnic minority medical systems, and gut microbiota be connected? To answer this question, we have given the ensuing viewpoints.

### Review of the literature

There are specific monographs on illness prevention and treatment for Chinese ethnic minority medicine. The many ethnic minority remedies used for the prevention and treatment of various diseases can be identified using ancient writings and documents. Presents a few examples in **Table 2**.

**Table 2:** Collection of common ethnic diseases and ethnic medicines recorded in the literature (partial display).

No.	Nationality	Disease	Ethnic medicines	Medicine efficacy	Ancient books
1	Tibetan	Coronary heart disease https://doi.org/10.3389/fcimb.2020.00373	*Ophiocordyceps sinensis* (*Cordyceps sinensis*) https://doi.org/10.1177/2047487318785996 Salidroside https://doi.org/10.1111/jcmm.12871 Chenxiang Powder https://doi.org/10.19540/j.cnki.cjcmm.20200224.201 Corydalis Herba https://doi.org/10.19540/j.cnki.cjcmm.20210129.401 Duoxuekang https://doi.org/10.1016/j.jep.2020.113629	Activating blood circulation, removing blood stasis and clearing the channels,clearing heart heat, tranquilizing mind, and inducing resuscitation	Month Wang Yaozhen Four-Volume Medical Code
2	Mongolian	Type 2 diabetes Https://doi.org/10.1007/s12275-021-0454-8	Saradma https://doi.org/10.19540/j.cnki.cjcmm.20200527.108 Cymbaria https://doi.org/10.19540/j.cnki.cjcmm.20191213.401 Heyi, Xila, and Badagan https://doi.org/10.1155/2021/5532069 Zhi Mu https://doi.org/10.1155/2022/9308598 Pueraria root https://doi.org/10.1142/S0192415X18500891	Benefiting Qi, promoting the production of body fluid and resolving turbidity	Four parts of manna
3	Uighur	Hyperuricemia Https://doi.org/10.1186/s12906-015-0644-1	Karapxa decoction https://doi.org/10.1186/s12906-015-0644-1 Abnormal Savda Munziq https://doi.org/10.1155/2012/863101 *Saussurea involucrata* https://doi.org/10.1016/j.jep.2015.06.033 *Apium graveolen L.* doi:10.1016/0378-8741(95)01291-5. *Cichorium glandulosum* Boiss. et Huet doi: 10.1007/s00299-005-0953-9.	Relieving dampness and water, relieving pain and lowering turbidity	Ancient Uyghur medical and Pharmaceutical books
4	Zhuang	Rheumatoid arthritis https://doi.org/10.1016/j.jep.2022.115325	Longzuantongbi granules https://doi.org/10.1016/j.jep.2022.115325 Zhuang-Gu-Fang https://doi.org/10.1155/2020/8164064 Blumea balsamifera DC. doi:10.13703/j.0255-2930.2018.03.005. Litsea cubeba (Lour.) Pers. doi: 10.1097/MD.0000000000022264. Ambrosia artemisiifolia. doi:10.13703/j.0255-2930.2018.03.005	Promoting blood circulation and removing blood stasis, removing dampness and relieving pain	Chinese Zhuang Medicine
5	Dai	Rheumatoid arthritis Hu Qian, Li Limei, Wang Wei & Tang Xiaohu. (2021). Analysis of the characteristics of Chinese Dai medicine in treating rheumatic fever Rheumatism and arthritis (12),11-15. Cardiovascular disease Chen Rong, Chen Qinghua & ZHENG Jin. (2017). Research progress of Dai medicine in prevention and treatment of heart disease Chinese Journal of traditional Chinese Medicine (10),4582-4584.	*Arundina graminifolia* *Mappianthus iodoides* Hand. Semen Cassiae Mountain turtle Https://doi.org/10.13862/j.cnki.cn43-1446/r.2017.21.034 Https://doi.org/doi:10.19540/j.cnki.cjcmm.20170217.003 Aspidopterys glabriuscula (Wall.) A. Juss. doi:10.4268/cjcmm20161628 Thela coccinea Plumbago rosea Hu, Qian, Li, Li-Mei, Wang, Wei & Tang, Xiao-Hu. 2011 A new species of the genus Phyllostachys (Hymenoptera, Braconidae) from China. (2021). Exploration of the medication characteristics of the Chinese Dai Medical Single Experiment Secret Formula for the treatment of rheumatic fever paralysis. Rheumatism and Arthritis(12),11-15.	Removing wind and relieving pain, relaxing tendons and activating blood circulation	Palm leaf classic
6	Yi	Type 2 diabetes, rheumatoid arthritis	Shekaqi https://doi.org/10.1002/CBDV.202200363. Yi Bu A Jie https://doi.org/10.1007/s11655-012-1177-9. Alangium chinense Https://doi.org/10.13862/j.cnki.cn43-1446/r.2020.05.013. Wosi https://doi.org/10.3389/fphar.2020.568585 Aralia chinensis L. doi: 10.1186/s13002-020-00400-5.	Clearing heat and dampness, resolving blood stasis and removing toxins, activating blood circulation and relieving pain	Qi Su Shu

### Experimental research

Innovative measurements, models, and designs are used in experimental medicine to investigate human subjects, prove the mechanisms at work in new pharmaceuticals, determine how best to differentiate successful drug candidates on the market, and stop the development of failing drug candidates. Because of the ambiguous validity and efficacy of novel targets and therapeutic candidates discovered by genomics, combinatorial chemistry, high-throughput screening, and the use of unreliable preclinical models, humans are the ultimate “model.” Because of advancements in clinical biomarkers, detailed examination of pharmacological effects and the nature of illness progression is becoming increasingly possible (Littman and Williams, 2005). Even if ethnic medicine is extensively practiced by people, a dearth of experimental research has muddled our understanding of how pharmaceuticals work, making it more difficult to adopt and popularize these drugs as well as to innovate their use. Developing Chinese ethnic medications can be accomplished by researching the association between the intestinal flora of the local ethnic minorities and the drugs used in various ethnic communities. This strategy has been used, for instance, in experimental studies investigating the mechanism of the Dai medicine “Ya Jie” in treating constipation and diarrhea, as well as studies investigating the effects of Zhuang moxibustion on irritable bowel syndrome (**Table 2**).

### Clinical investigation

All medical decisions must be supported by reliable scientific evidence (Szajewska, 2018). To investigate the herbal medicine intervention mechanism in ethnic minority areas based on the intestinal microorganisms, it is important to understand the types of intestinal microorganisms in ethnic minorities in China and the relationship between the intestinal microorganisms in members of ethnic minorities and the occurrence of diseases. Investigating whether the prevalence of cardiovascular diseases in Yi and Tibetan regions is related to the variations in intestinal flora between Yi and Tibetan nationalities, as well as the effects of Yi and Tibetan medicines used in the two regions, on the gut microbiota, would be one example of such an investigation.

**Figure 8:**
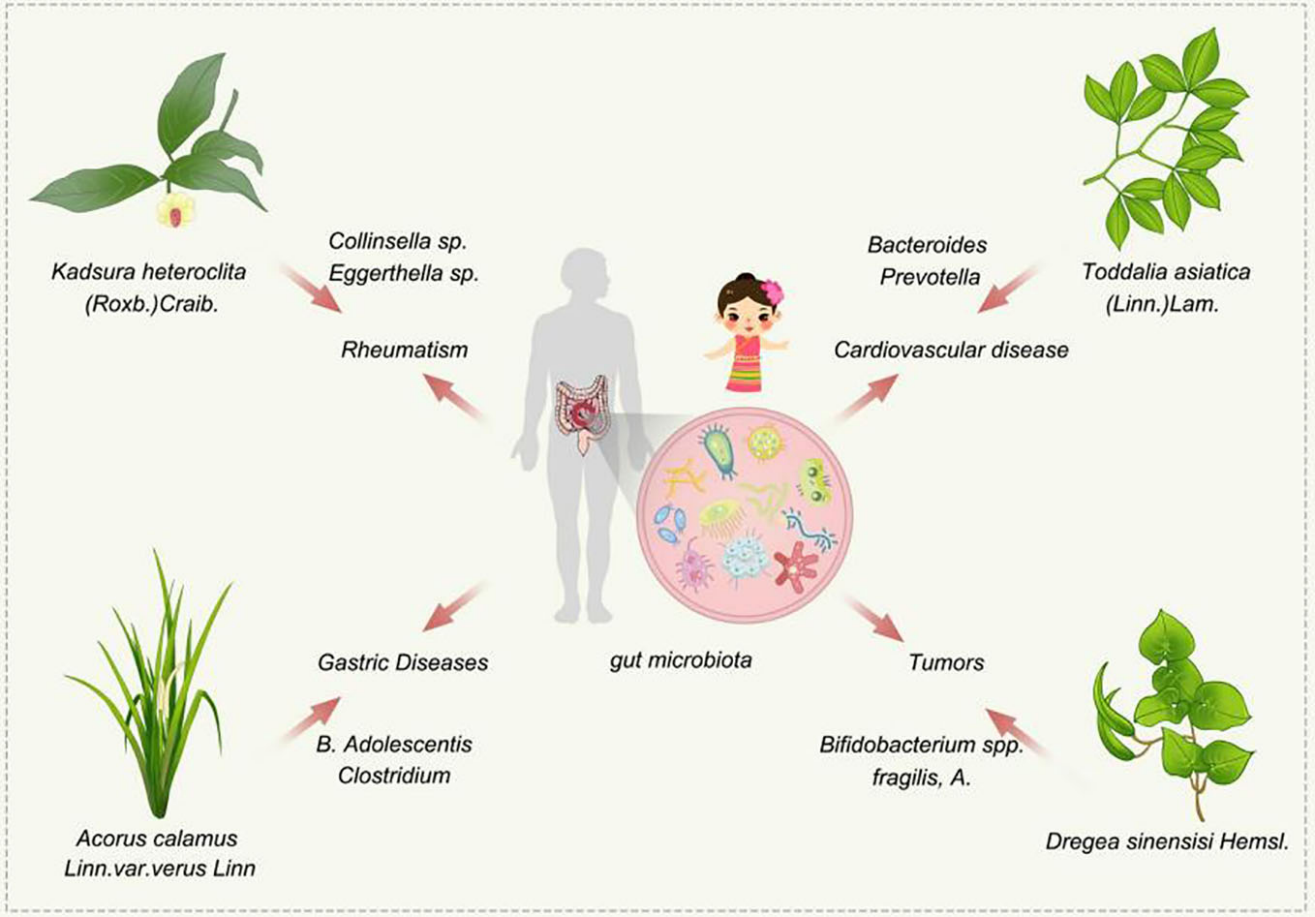
Schematic diagram of Dai medicine intervening in endemic diseases of Dai population through gut microbes.

For example, Dai people are prone to stomach diseases, rheumatism, tumors, and cardiovascular diseases due to differences in gut microbiota and other factors, and some Dai medicines (*Kadsura heteroclita* (Roxb.)Craib.,*Toddalia asiatica*(Linn.)Lam., *Dregea sinensisi* Hemsl.,*Acorus calamus Linn.var.verus* Linn)in Dai medicine are commonly used to treat these diseases, so the mechanism of action of drug intervention in endemic diseases that are highly prevalent can start from the gut microbiota and find the mechanism of action of Dai medicines for endemic diseases based on gut microbiota (**Figure 8**).

## Conclusions and perspectives

There are currently more than 220 countries and regions in the world, with more than 2,000 different ethnic groups. People from many different ethnic groups have amassed a lot of medical knowledge and practical experience over the course of continual upheaval and peaceful coexistence with nature. As a result, several traditional medical practices (and systems) have emerged and grown around the world (Yuan and Xiao, 2013). TCM includes Chinese minority medicine, which is a significant component, as an alternative medical practice. One of the things affecting gut microbes is racial disparities. This area of research is receiving more attention and is being acknowledged by numerous journals with high impact factors. China has a complex gut microbiota and a variety of endemic diseases due to its multiethnic population. To treat and prevent endemic diseases, each ethnic group utilizes its unique methods and medications. We provide ways of thinking and tactics based on these ideas. Literature reviews, fundamental studies, and clinical studies can all be used to assess the ethnic medicine’s approaches to illness prevention and treatment. We think that in the future, it will progressively become clearer how Chinese minority medicine regulates the entire field of intestinal health. Future study on the constituents of traditional medicines and the targets of their impacts on gut microbes is something we intend to expand. It is crucial to carry out further research on how traditionally used medicines can be helpful.

## Data availability statement

The original contributions presented in the study are included in the article/Supplementary Material. Further inquiries can be directed to the corresponding authors.

## Author contributions

RC and Q-HC did the manuscript drafting, Z-YD and X-HD did the language revision, and JZ guided the revision. All authors contributed to the article and approved the submitted version.

## Funding

Supported by Yunnan Provincial Science and Technology Department-Applied Basic Research Joint Special Funds of Chinese Medicine; No.2017FF117-019, National Social Science Fund; No.21BMZ135, Yunnan Key Laboratory of Dai and Yi Medicines, Yunnan humanities and social sciences research project.

## Acknowledgments

We would like to thank Yunnan Key Laboratory of Dai and Yi Medicines support for ethnic medicine research.

## Conflict of interest

The authors declare that the research was conducted in the absence of any commercial or financial relationships that could be construed as a potential conflict of interest.

## Publisher’s note

All claims expressed in this article are solely those of the authors and do not necessarily represent those of their affiliated organizations, or those of the publisher, the editors and the reviewers. Any product that may be evaluated in this article, or claim that may be made by its manufacturer, is not guaranteed or endorsed by the publisher.
